# Investigating the effect of intra-operative infiltration with local anaesthesia on the development of chronic postoperative pain after inguinal hernia repair. A randomized placebo controlled triple blinded and group sequential study design [NCT00484731]

**DOI:** 10.1186/1471-2482-7-22

**Published:** 2007-11-06

**Authors:** Philipp Honigmann, Henning Fischer, Anita Kurmann, Laurent Audigé, Guido Schüpfer, Jürg Metzger

**Affiliations:** 1 Department of Surgery, Kantonsspital Luzern, Luzern, Switzerland; 2 AO – Clinical Investigation and Documentation, Davos, Switzerland; 3 Department of Anaesthesiology, Kantonsspital Luzern, Luzern, Switzerland

## Abstract

**Background:**

Inguinal hernia repair is one of the most frequently performed procedures in Switzerland (15'000/year). The most common complication postoperatively is development of chronic pain in up to 30% of all patients irrespective of the operative technique.

**Methods/Design:**

264 patients scheduled for an inguinal hernia repair using one of three procedures (Lichtenstein, Barwell and TEP = total extraperitoneal hernioplasty) are being randomly allocated intra-operatively into two groups. Group I patients receive a local injection of 20 ml Carbostesin^® ^0.25% at the end of the operation according to a standardised procedure. Group II patients get a 20 ml placebo (0.9% Saline) injection. We use pre-filled identically looking syringes for blinded injection, i.e. the patient, the surgeon and the examinator who performs the postoperative clinical follow-ups remain unaware of group allocation. The primary outcome of the study is the occurrence of developing chronic pain (defined as persistent pain at 3 months FU) measured by VAS and Pain Matcher^® ^device (Cefar Medical AB, Lund, Sweden).

The study started on July 2006. In addition to a sample size re-evaluation three interim analyses are planned after 120, 180 and 240 patients had finished their 3-months follow-up to allow for early study termination.

**Discussion:**

Using a group sequential study design the minimum number of patients are enrolled to reach a valid conclusion before the end of the study.

To limit subjectivity, both a VAS and the Pain Matcher^® ^device are used for the evaluation of pain. This allows us also to compare these two methods and further assess the use of Pain Matcher^® ^in clinical routine.

The occurrence of chronic pain after inguinal hernia repair has been in focus of several clinical studies but the reduction of it has been rarely investigated. We hope to significantly reduce the occurrence of this complication with our investigated intervention.

**Trial Registration:**

Our trial has been registered at ClinicalTrials.gov. The trial registration number is: [NCT00484731].

## Background

Inguinal hernia repair is one of the most frequently performed procedures in Switzerland (about 15'000 cases per year) [1]. The most common complication postoperatively is development of chronic pain in up to 30% of patients irrespective of the operative technique [2-7]. Callesen et al. [3] found the persistence of pain at 1 and 4 weeks after surgery to be a predictive factor on the development of chronic pain. Courtney et al. [8] concluded that chronic pain persists in most patients who report pain up to 3 months after hernia repair. Surgical procedures like the ilioinguinal neurectomy recently described by Mui et al. [9] lead to a significant reduction of chronic pain but also to numbness and discomfort in the groin region. Whereas Ravichandran et al. [10] in contrast showed no significance of the elective division of the ilioinguinal nerve.

In addition to the problems caused by hernia surgery the objective evaluation of the postoperative pain is limited. The Visual Analogue Scale (VAS) is often used in similar study settings and is well known as "gold standard" for eliciting pain. However Lundeberg et al. [11] reported the VAS is a questionable instrument for the measurement of pre- and postoperative pain because it remains very subjective and is based on varying previous pain experience. Hence the VAS has a misleading precision because of an imprecise individual determination of the VAS of about ± 20 mm (DeLoach et al.) [12].

An alternative option for measuring pain is the recently developed Pain Matcher^® ^by Cefar Medical AB, Lund, Sweden. This perceptual matching device uses electrical stimulation against which the patient has to match the perceived pain with nonvisualized endpoints. It seems to be more objective and reduces recording bias. The device has been tested for safety and reliability in several clinical trials [13-16] and has not been used so far in the field of hernia surgery.

The use of local compared to general anaesthesia in hernia surgery has only been described by O'Dwyer et al. [17] not investigating the outcome on the development of chronic pain after hernia repair. A positive effect of postoperative single shot or continuous infiltration with local anaesthetics on acute postoperative pain has been described by several authors [18-20].

We designed a randomized placebo controlled triple blinded trial to investigate the effect of an additional intra-operative infiltration with local anaesthesia on the development of chronic postoperative pain. Because of incertainty of data used for sample size estimation, a sequential design data analysis for early study stopping along with revision of sample size is applied. As Aasvang and Kehlet [21] pointed out there is an urgent need for the treatment opportunities and assessment of chronic pain after herniorrhaphy. We hope to contribute with this study to this common problem after hernia repair.

## Methods/Design

**Figure 1:**
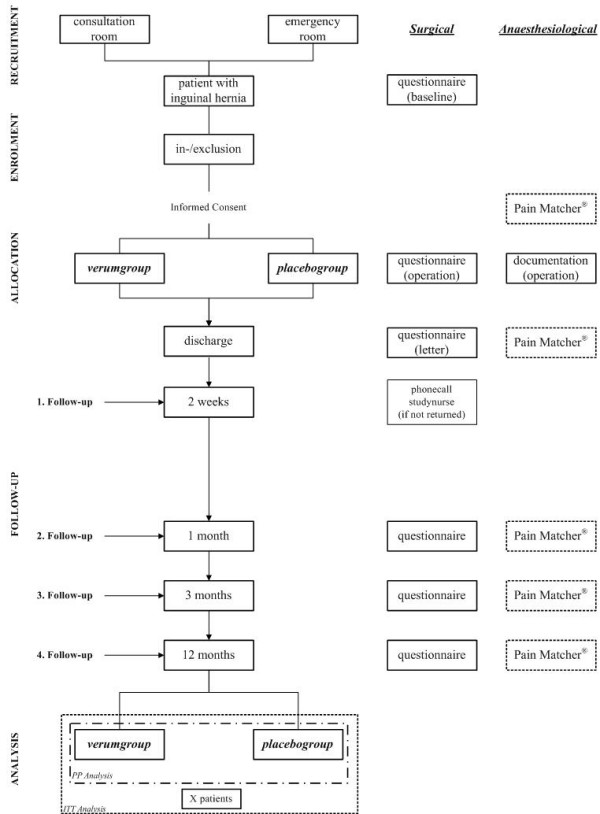
patient flow according to the revised CONSORT-statement.

Patients are recruited in the outpatients and the emergency department. If they meet the inclusion criteria (see next section), informed consent is obtained and the operation is planned. According to our randomization process the patient is automatically either allocated to the verum or control group by the sequence of the consecutive numbered and block randomized syringes. An overview of the patient recruitment and follow-up is presented in Figure 1.

### Inclusion and exclusion criteria

Adult patients (≥ 18 years) with primary or recurrent single or double sided symptomatic but not incarcerated inguinal hernias with an elective hernia repair are included. No other interventions (i.e. umbilical hernia) are allowed. Written informed consent needs to be obtained.

Patients with legal incompetence, pregnant and nursing women, patients with presence or history of active malignancy or systemic diseases, under immunosuppressive treatment, with systemic or severe local inflammation or infection, with wound healing disorders and with physical or mental incapacity, which makes it impossible to obtain informed consent are excluded. As pacemakers interfere with the electrical stimulation of the Pain Matcher^® ^and vice versa patients with pacemakers or other implanted electrical devices were also excluded.

### Surgical Methods

Allowed standardized surgical procedures for the hernia repair are Lichtenstein [22,23], Barwell [24] and TEP [25]. These procedures for the hernia repair are performed directly or under the supervision by used to test the efficacy of the local infiltration. The operation is performed by a general surgery consultant or, a senior resident or under supervision of one of them. The hierarchical position and individual surgeon experience in the field of regarding inguinal hernia repair (i.e. number of performed operations) is documented.

### Anaesthesiological Methods

**Figure 2:**
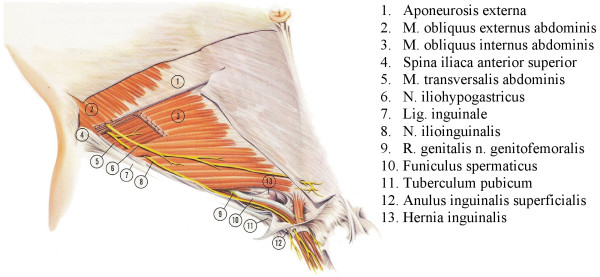
anatomy of the groin region.

**Figure 3:**
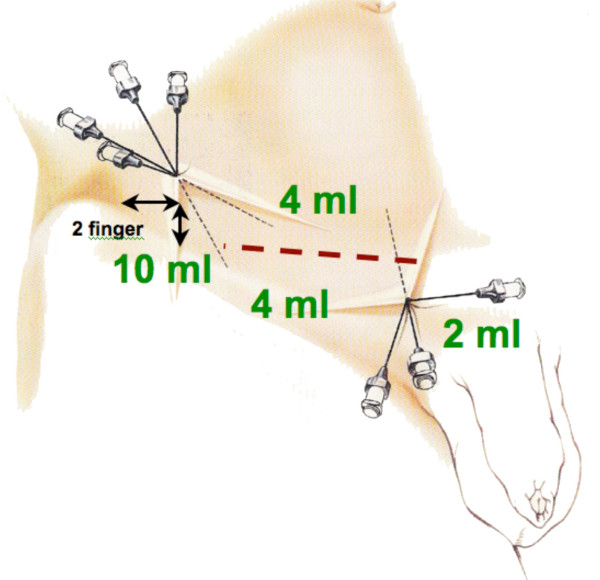
guide for the injection of the verum/placebo.

After the repair of the inguinal hernia the verum *Bupivacain *(Carbostesin^® ^0.25%) or the placebo *Saline *(0.9 %) is injected close to the nerves of the groin region following a standardized procedure (Figure 2). The injection starts 2 fingers below and 2 fingers medial the spina iliaca anerior superior on the lateral end of the incision (Figure 3). Ten ml of the substance will be injected fan-shaped laterally and 4 ml medially of the cranio-lateral puncture. The medio-caudal puncture is located directly above the pubic tubercle on the medial end of the incision. 4 ml of the substance will be fan-shaped injected lateral and 2 ml medial of the puncture.

### Randomization & Blinding

The randomization based on a computer-generated block randomization sequence is performed in 1:1 ratio between the investigational and the control arm. The pharmacy of our hospital provides equal looking syringes of placebo and verum numbered according to the randomization sequence that is kept confidential. Patient, surgeon and the doctor performing examinations during follow-up visits will not know about treatment allocation for each patient, which leads to this unique triple blinded study design.

The randomization code will be broken only in the following circumstances:

- for all patients at the completion of the final data analyses

- for individual patients following the occurrence of adverse events requiring immediate knowledge of treatment allocation for the safety of the patient.

### The Pain Matcher Device

**Figure 4:**
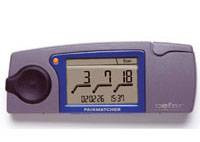
Pain Matcher^®^

The Pain Matcher^® ^(Cefar Medical AB, Lund, Sweden; Figure 4) is a perceptual matching device unit that gives constant current stimulation which is controlled by a microprocessor that provides rectangular pulses with a frequency of 10 Hz and an amplitude of 10 mA. It is programmed to give a constant current stimulation despite variable skin resistance (e.g., influenced by sweating and anxiety of the subject) up to 13 kΩ. The intensifying of stimulation is achieved by successively increasing the pulse width from 0 to a possible maximum of 450 μs in increments of 7.5 μs, up to a total of 60 steps. The electrical charge per second is extremely low and varies through the different steps from 1.5 to 45 μC. The reached value (0–100) is directly related to the pulse width and is displayed on a liquid crystal display screen. The contact surface area, and hence the resulting current density, is ensured by a certain minimum finger pressure against the electrode; this is achieved by instructing the patient to hold the electrode box between the thumb and the index finger in a horizontal position requiring a certain minimum, predetermined pressure. A further increase in electrode pressure does not improve the functionality of the Pain Matcher^®^.

### Primary Outcome

According to Courtney et al. [8] who concluded that chronic pain persists in most patients who report persistent pain at 3 months after hernia repair, we chose the occurrence of chronic pain (persistent pain at 3 months FU measured by VAS and Pain Matcher^®^) in the operated groin region for our primary outcome.

### Secondary Outcomes

#### Level of Pain

- Pain Matcher^®^

- VAS

- Areas of hyperalgesia, hypaesthesia

#### Hospitalization

- Length of stay (days)

- ASA-Classification

- Beginning of mobilisation (days)

- Removal of drainage (days)

#### Function

- Return to work or normal activity (days and %)

- Quality of life (SF36)

Amounts of follow-up and recorded parameters are summarized in Table 1.

**Table 1:** Follow-up and recording of data

	**Baseline**		**FU 1**	**FU 2**	**FU 3**	**FU 4**
	Consultation	Intra-/post-OP	2 weeks	1 month	3 months	12 months
**General data**						
- Patients data						
- Informed consent						
**Anaesthesiological data**						
**Surgical intervention**						
**Edverse events**						
Primary outomes:						
- VAS						
- Pain Matcher^®^						
Secondary outcomes:						

### Hypothesis

We hypothesise a 50% reduction of the occurrence of postoperative chronic pain after 3 months in the intervention group receiving intra-operative infiltration with local anaesthetic after an inguinal hernia repair compared with patients in the control group receive placebo infiltration.

### Sample size & power calculation

Required sample sizes and interim analysis data for both groups were estimated using the computer programme East^® ^Version 4 (Cytel Inc., 675 Massachusetts Ave. Cambridge, MA02139 USA). Sample size estimation was based on a risk of occurrence of chronic pain without local anaesthetic infiltration of 30% in patient having inguinal hernia surgery (Poobalan et al. [4]).

Considering a 0.05 two-sided significance level, a power of 80%, a detectable risk reduction of 50% and an allocation ratio of 1:1, 120 patients per group were estimated.

Assuming a dropout rate of 10% (declining, exclusion, loss to follow-up, etc.) in the study, ***132 patients per group***(264 patients) are going to be enrolled.

### Group sequential design and interim analysis

To allow for early stopping of the trial, a group sequential analysis is performed. The first of three interim analyses will be performed as soon as 120 patients (60 per group) have had their 3-month follow-up. Further interim analyses are planned after 180 and 240 patients (final analysis).

We will use a χ^2^-test to test the null-hypotheses. The analysis will be performed on the 'intention-to-treat' basis, i.e. patients will be analyzed in the groups to which they were randomized.

### Stopping rules

The randomization code will not be broken for the interim analyses, unless a stopping rule is met. At each interim analysis, the following stopping rules will apply:

1. A χ^2^-test demonstrates that one treatment is superior over the other (i.e. rejection of the null-hypothesis) OR,

2. If statistical significance cannot be likely reached with the maximum allowable number of patients for the study, which is set to 200 per arm (i.e. acceptance of the null-hypothesis).

**Figure 5:**
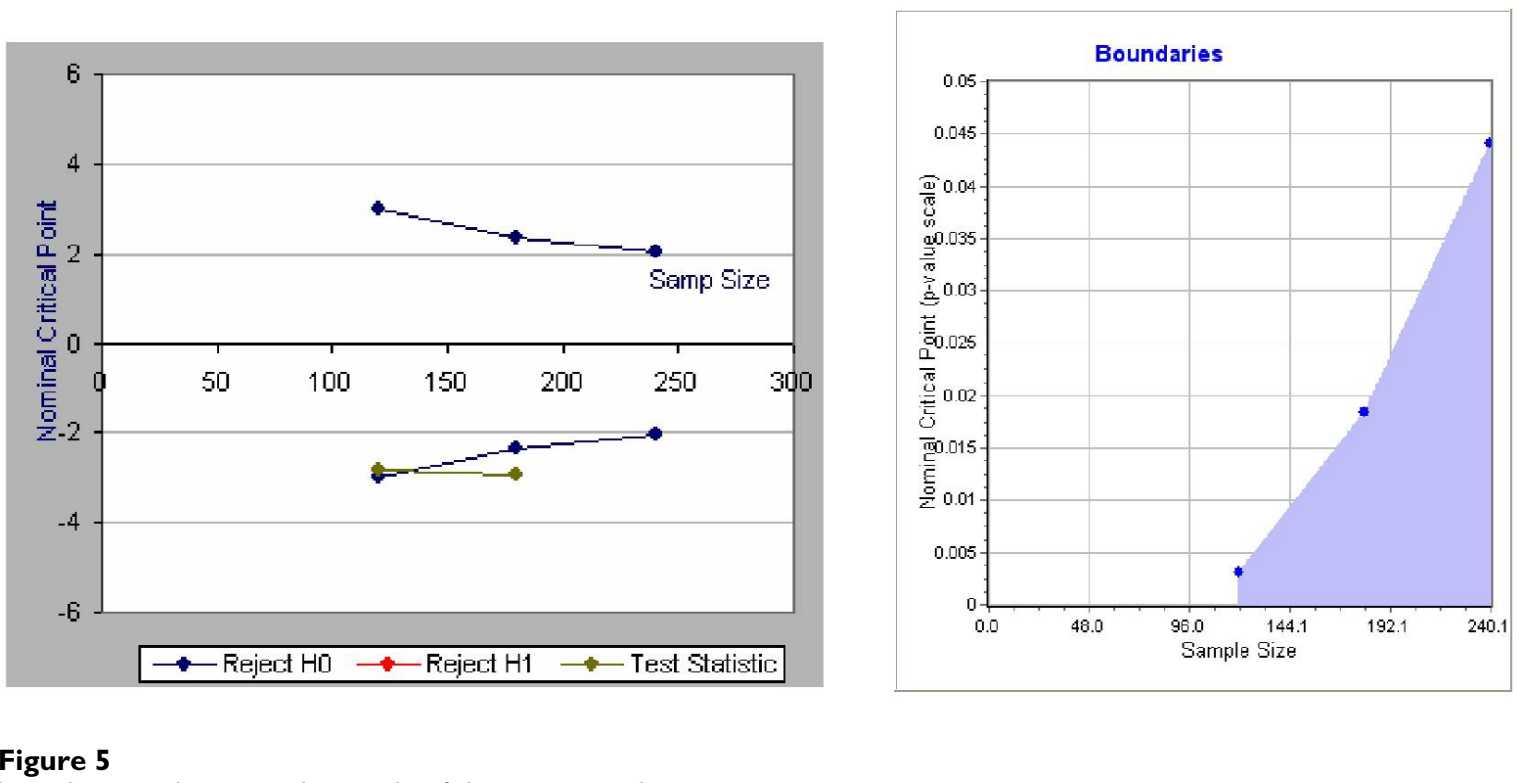
boundaries and estimated example of the interim analysis.

Table 2 shows the threshold z-values for the χ^2^-test at each interim analysis. Boundaries for the interim analyses are calculated on the model developed by O'Brien & Fleming [26-32] (Figure 5).

**Table 2:** Threshold z-values for the χ^2^-test at each interim analysis

**Interim analyses**	**Number of patients**	**Reject H_0 _if z ≥ **	**Accept H_0 _if z <**	**Reject H_0 _if p ≤**
1	120	-2.9626	2.9626	0.0031
2	180	-2.359	2.359	0.0183
3 = final analysis	240	-2.0141	2.0141	0.044

If a stopping criterion has not been reached, the trial will continue until the next interim analysis. If the trial is stopped after an interim analysis, all patients already enrolled in the trial will be followed up to the last planned visit.

### Sample size re-estimation

Independent of the sequential design, a sample size re-estimation will be conducted before the first interim analysis, and the number of patients required for each interim analysis will be adapted accordingly. The study will be continued until the next interim analysis or terminated if more than 200 patients per arm are additionally needed to show a significant result.

All interim analyses will be performed by an independent organization without the knowledge of the randomization code. The revision of the sample size will be made available to the investigators, if applicable. No other results will be provided to the investigators, unless a stopping rule is fulfilled and the study has to be terminated.

### Ethical approval

The study was approved by the local ethical committee of the Kanton Luzern on the 1^st ^of May 2006 and the first amendment on the 26^th ^of February 2007 (approval number 585).

## Discussion

Chronic pain after inguinal hernia repair is a common complication that needs to be addressed. This randomized placebo controlled triple blinded designed trial should provide conclusive results regarding the effectiveness of intra-operative use of local anaesthesia in mitigating this problem.

Using a group sequential study design, a minimum number of patients will be recruited until interim results suggest continuing the study became unethical. This approach has been described first by Pocock [33,34] who provided methodological guidelines already in the late 70's and early 80's. O'Brien and Fleming [26] described tests with conservative stopping boundaries for interim analyses with a decision rule. Methods determining the sample size needed for the group sequential test to attain the desired power were described by Fleming, Harrington and O'Brien [35] and by Wang & Tsiatis [36]. As this study design should be attractive to clinical researchers reducing the number of unnecessarily involved human being in clinical trials it has been used rarely since the mentioned publications in the 80's! A search in Pubmed [37] returns 439 randomized controlled trials using a group sequential design out of 215'249 since 1980, only 2 ‰ in the last 27 years!

We are using the VAS and Pain Matcher^® ^for the evaluation of pain which allows us to compare these two methods and confirm the good results of the recently published studies [13-16] using the Pain Matcher^® ^in clinical routine.

Publishing this study protocol we contribute to good, honest and ethically correct clinical research already called for in 1999 by Chalmers and Altman [24]. This step should be included in official guidelines like the CONSORT-Statement and other authors should be convinced to publish their study protocols fighting against poor and ethically unfair research in medicine!

## List of abbreviations

ASA american society of anaestesiologists

CONSORT Consolidated Standards of Reporting Trials

FU follow up

RCT randomised controlled trial

SF36 short form 36

TEP total extraperitoneal hernioplasty

VAS visual analog scale

## Competing interests

The authors declare that they have no competing interests especially not with the companies mentioned in this publication.

## Authors' contributions

PH was responsible for the methodological background, the final design of the trial, drafted the clinical investigation plan and the manuscript. HF conceived of the main study idea, drafted the manuscript as well and was responsible for the coordination. AK participated in the design and coordination. GS conceived of the anaesiological method section. LA is responsible for the sequential design and helped to draft the CIP and the manuscript. JM is the principal clinical investigator and responsible for the whole project especially the surgical background. All authors read and approved the final manuscript.

## Pre-publication history

The pre-publication history for this paper can be accessed here:


